# What Does Brain Response to Neutral Faces Tell Us about Major Depression? Evidence from Machine Learning and fMRI

**DOI:** 10.1371/journal.pone.0060121

**Published:** 2013-04-01

**Authors:** Leticia Oliveira, Cecile D. Ladouceur, Mary L. Phillips, Michael Brammer, Janaina Mourao-Miranda

**Affiliations:** 1 Department of Neuroimaging, King’s College London, London, United Kingdom; 2 Instituto Biomédico, Universidade Federal Fluminense, Niterói, Brazil; 3 Department of Psychiatry, Pittsburgh University, Pittsburgh, Pennsylvania, United States of America; 4 Department of Psychological Medicine, Cardiff University, Cardiff, United Kingdom; 5 Department of Computer Science, University College London, London, United Kingdom; The University of Melbourne, Australia

## Abstract

**Introduction:**

A considerable number of previous studies have shown abnormalities in the processing of emotional faces in major depression. Fewer studies, however, have focused specifically on abnormal processing of neutral faces despite evidence that depressed patients are slow and less accurate at recognizing neutral expressions in comparison with healthy controls. The current study aimed to investigate whether this misclassification described behaviourally for neutral faces also occurred when classifying patterns of brain activation to neutral faces for these patients.

**Methods:**

Two independent depressed samples: (1) Nineteen medication-free patients with depression and 19 healthy volunteers and (2) Eighteen depressed individuals and 18 age and gender-ratio-matched healthy volunteers viewed emotional faces (sad/neutral; happy/neutral) during an fMRI experiment. We used a new pattern recognition framework: first, we trained the classifier to discriminate between two brain states (e.g. viewing happy faces vs. viewing neutral faces) using data only from healthy controls (HC). Second, we tested the classifier using patterns of brain activation of a patient and a healthy control for the same stimuli. Finally, we tested if the classifier’s predictions (predictive probabilities) for emotional and neutral face classification were different for healthy controls and depressed patients.

**Results:**

Predictive probabilities to patterns of brain activation to neutral faces in both groups of patients were significantly lower in comparison to the healthy controls. This difference was specific to neutral faces. There were no significant differences in predictive probabilities to patterns of brain activation to sad faces (sample 1) and happy faces (samples 2) between depressed patients and healthy controls.

**Conclusions:**

Our results suggest that the pattern of brain activation to neutral faces in depressed patients is not consistent with the pattern observed in healthy controls subject to the same stimuli. This difference in brain activation might underlie the behavioural misinterpretation of the neutral faces content by the depressed patients.

## Introduction

The ability to identify facial emotional expressions in individuals is essential to functioning in social networks because the perception of emotional faces can influence the production and regulation of affective states subserving adaptive social behaviour [1]. Affective states and mental illness are associated with altered processing of emotional facial expressions. In fact, abnormal facial emotion processing, and abnormal neural activation to emotional facial expressions, has been shown in a range of psychiatric conditions, including major depression [2], [3]. For instance, Surguladze et al. [4] found increased neural responses in subcortical areas to sad but not happy expressions in depressed patients compared with healthy controls. Other studies have reported that depressed patients had greater amygdalar and ventral striatum activation to sad faces [5] and reduced activation to happy faces in the regions of the putamen, hippocampus, and ventral striatum compared with healthy controls [6]. These findings provide support for the presence of mood-congruent processing bias in depression, (i.e. hyperactivation to negative and hypoactivation to positive stimuli, particularly in the amygdala, insula, parahippocampal gyrus, fusiform face area, and putamen (see [7]). More recent studies employing functional connectivity analyses have also reported abnormalities in prefrontal-subcortical circuitry. For instance, using dynamic causal modeling (DCM), Almeida et al. [8] showed reduced left-sided top-down orbitofrontal cortex-amygdala effective connectivity during happy and sad facial brain processing in depressed subjects compared to healthy controls. In the same vein, connectivity studies using resting-state fMRI in major depression have reported abnormalities in fronto-limbic connectivity [9], [10]. Taken together studies of facial emotion processing may provide important information regarding abnormalities of regional brain functioning in major depression and this abnormal processing may help in the prediction or monitoring of response to treatment in major depression [1].

The studies described above relate to processing of emotional face expressions such as angry or sad faces. Less attention, however, has been given to patterns of abnormal neural activation to *neutral* faces. Behaviourally, depressed patients are less accurate at recognizing neutral expressions compared with healthy controls. Specifically, they are more likely to misinterpret neutral faces as sad, and happy faces as neutral, suggesting a negative bias in these patients [11]. Furthermore, they are slower to respond to neutral compared with emotional facial expressions [11], [12]. These behavioral findings suggest that major depression may also involve abnormalities in neutral face processing but to date few studies have investigated the neural correlates of neutral face processing in depressed patients. Such data would advance our understanding about emotional processing in depression and help elucidate further potential biomarkers of emotion face processing that may contribute to the pathophysiology of major depression.

Recently, pattern recognition techniques have been applied to detect patterns of brain activation that distinguish between cognitive states (e.g. [13], [14], [15]) or between healthy individuals and patients with psychiatric or neurological disorders (e.g. [16], [17], [18], [19]). Furthermore, it has recently been shown that pattern recognition can help to discriminate healthy low-risk control adolescents from healthy adolescents at genetic risk of future psychiatric disorders, indicating that this approach can help to identify which individuals at risk are at true risk of developing future Axis I disorders [20]. In these applications brain scans are treated as spatial patterns and statistical learning methods are used to identify statistical properties of the data that discriminate between groups of subjects. Once the discriminative pattern is found, it can be used to classify individuals, case by case, into groups based on their pattern of brain activation. The most common statistical approach for analyzing fMRI data is the General Lineal Model (GLM, [21]), which treats every voxel in the brain independently and extract measures of interest from them, such as the average response during a particular experimental condition or for a specific population. Another important advantage of pattern recognition approaches is that the predictions are made based on the information encoded on the whole pattern rather than in individual brain voxels (i.e. they are multivariate), which can lead to increased sensitivity over voxel-wise analysis methods [15].

In the present study, we investigated whether the misclassification described behaviourally for neutral faces also occurred when classifying patterns of brain activation to neutral faces for patients with major depression. Using healthy control samples as reference we tested whether the discriminating pattern between brain activation to emotional versus neutral faces (based on the healthy subjects) could be used to classify the brain activation of the patients to the same stimuli. For that we used two fMRI data sets: (1) Nineteen medication-free patients with depression and 19 healthy volunteers and (2) Eighteen depressed individuals and 18 age and gender-ratio-matched healthy controls.

Our novel pattern recognition framework consisted of two phases: first, we trained the classifier to discriminate between two brain states (e.g. viewing happy faces vs. viewing neutral faces) using data only from healthy controls (HC). Second, we tested the classifier using the patterns of brain activations of a patient and a healthy control subject to the same stimuli. The rationale of this procedure was to investigate how similar would be the patients’ brain activation pattern relative to activation in a healthy comparison subject. In other words the idea was to consider the healthy brain pattern as a “reference pattern” and to investigate if the discriminating pattern between emotional versus neutral stimuli based on the healthy subjects could be applied to classify the brain activation of patients. A misclassification of the patients’ pattern of brain activation would represent an inconsistency with respect to the healthy patterns. Therefore, this approach is very different from those performed in the previous studies in which the two populations (healthy controls and depressed patients) were directly compared. Specifically, we applied a standard leave-one-out cross-validation procedure in the healthy control group, i.e. we train the classifier with all but one control subject to discriminate between patterns of brain response to emotional and to neutral faces. We then tested the classifier using data from the healthy control left out and a matched depressed patient. This procedure was repeated, each time leaving a different control subject out for testing. We used Gaussian Process Classifier (GPC) as it provides a predictive probability to the test samples. The predictive probability measures the classifier’s confidence about the class membership of a test example. If the predictive probability is close to 0.5, it means the classifier is not very confident which indicates that the pattern of brain activation might be ambiguous and/or different from the patterns used to train the classifier. Our aim was to investigate if the pattern of brain activation of patients would be classified with the same confidence level as the HC patterns. In this case a lower confidence level would indicate that the pattern of brain activation in depressed patients is not consistent with the pattern of brain activation in healthy subjects. We used patterns of brain activation to prototypical emotional (100% sad or 100% happy) vs. neutral faces to maximize the differences between the patterns used to train the GPC. Based on behavioural findings [11], [12], we expected to observe less discrimination between these patterns for depressed patients than for healthy subjects. Finally, we compared the classifier’s predictive probabilities for emotional and neutral faces classification between healthy controls and depressed patients.

## Methods

### Subjects

#### Sample 1

Nineteen participants (13 women; age range: 29–58 years, see Table 1) meeting DSM-IV criteria for major depressive disorder according to the Structured Clinical Interview for DSM-IV Axis I Disorders [22] and clinical interview with a psychiatrist. The severity of depression was evaluated with the 25-item Hamilton Rating Scale for Depression [23]. All patients were free of psychotropic medication for a minimum of 4 weeks at recruitment. Nineteen healthy comparison subjects (11 women), matched by age and intelligence quotient (IQ), with no history of any psychiatric disorder, neurological disorder, or head injury resulting in a loss of consciousness were recruited. All participants provided written, informed consent. The project was approved by the Ethics Research Committee, Institute of Psychiatry,London, England.

**Table 1:** Demographic Features and Severity of Depression.

	Sample 1		Sample 2	
	Depressed Patients	Healthy Control Subjects	Depressed Patients	Healthy Control Subjects
	*n* _ 19	n _ 19	n _ 18	*n* _ 18
Mean Age (years)	43.2 (8.8)	42.8 (6.7)	31.9 (9.2)	29.8 (9.1)
Gender (m/f)	6/13	8/11	1/17	3/15
HRSD 25*	21.1 (2.3)		22.8 (7.5)	
Medication Load			1.4 (1.1)	

* HRSD-25∶25-item Hamilton Rating Scale for Depression.

#### Sample 2

Eighteen currently depressed patients with recurrent unipolar depression based on standardized diagnostic criteria for these illnesses [24] (age range 18–54 years, see Table 1). The severity of depression was also evaluated with the 25-item Hamilton Rating Scale for Depression [23]. Eighteen healthy control individuals matched by gender (age range) with no previous psychiatric history (based on SCID-P criteria) or psychiatric history in first and second-degree relatives also participated in the study All participants provided written, informed consent after explanation of the nature and possible consequences of the study. The study was approved by the University of Pittsburgh Institutional Review Board.

### fMRI Tasks

In sample 1, the event-related fMRI experiment included ten faces (5 male faces) from a standardized series of facial expressions of sadness [25] that were morphed to represent three emotion intensities (neutral, 50% sad and 100% sad). For the fMRI paradigm, facial stimuli and baseline trials (crosshair fixation) were presented in random order. Each facial stimulus was presented twice at each intensity of sadness and each trial was presented for 3 seconds. The interval was randomly varied according to a Poisson distribution with mean inter-trial interval of 5 seconds. For each facial trial, subjects were asked to indicate the gender of the face by lateral movement of a joystick; no hand movement was required in response to the baseline trial [5].

In sample 2, all individuals participated in a 6-minute event-related experiment [4]. The experiment involved viewing 60 morphed facial expressions to depict expressions ranging from neutral to intense happy (neutral, 50% happy and 100% happy). Each facial expression was presented for 2 seconds, with an inter-stimulus interval (ISI) of variable duration, varied according to a Poisson distribution (mean ISI = 4.9 s). Participants were asked to label the emotion of each face by moving either the index (emotional faces) or middle finger (neutral faces) of the right hand to ensure that attention was directed to the emotional content of the face.

### fMRI Data Acquisition

In sample 1, neuroimaging data were collected using a 1.5-T IGE LX System (General Electric, Milwaukee, Wisconsin) BOLD functional images were then acquired with a gradient echo EPI sequence covering 16 axial slices (7 mm thick, 0.7 mm gap; TR/TE = 2000/40 msec, in-plane resolution 3×3 mm).

In sample 2, neuroimaging data were collected using a 3.0 Tesla Siemens Allegra MRI scanner. BOLD functional images were then acquired with a gradient echo EPI sequence covering 33 axial slices (3 mm thick, 0 mm gap; TR/TE = 2000/25 msec, FOV = 24 cm, in-plane resolution 3×3 mm).

### fMRI Data Analysis

#### fMRI Data preprocessing and GLM analysis

In sample 1, data pre-processing was performed using standard procedures in SPM2. The fMRI data were realigned to remove residual motion effects, transformed into standard space using EPI template, and smoothed in space using an 8 mm Gaussian filter (full-width at half maximum [FWHM]). For each subject a GLM model was constructed in SPM2 with the three emotion intensities (neutral, 50% sad and 100% sad) entered in the design matrix as separate regressors in an event-related design with fixation cross as the baseline. Trials were modelled using the Canonical Hemodynamic Response Function in SPM2.

In sample 2, data pre-processing was performed using standard procedures in SPM5. The fMRI pre-processing procedure was similar to sample 1, but the functional data for each participant were first corrected for differences in acquisition time between slices and then were realigned to remove residual motion effects. Furthermore, the fMRI data were transformed into standard space using EPI template and were spatially smoothed with a Gaussian kernel of 6-mm full-width at half-maximum. Similar to sample 1, for each subject a GLM model was constructed in SPM5 with the three emotion intensities (neutral, 50% Happy and 100% Happy) entered in the design matrix as separate regressors in an event-related design with fixation cross as the baseline. Movement parameters from the realignment stage were entered as covariates of no interest to control for subject movement. Trials were modelled using the Canonical Hemodynamic Response Function in SPM5.

#### Medication load

In sample 2, we used a strategy for measuring total medication load in depressed patients [26], [27] by coding the dose of each antidepressant, mood-stabilizer, antipsychotic and anxiolytic (benzodiazepine) medication as absent (0), low (1) or high (2) dose. For antidepressants and mood-stabilizers we converted each medication into low- or high-dose groupings using a previously employed approach. Patients on levels 1 and 2 of these criteria were coded as low-dose, those with levels 3 and 4 as high-dose. We added a no-dose subtype for those not taking these medications. We converted antipsychotic doses into chlorpromazine dose equivalents, and coded as 0, 1 or 2, for no medication, chlorpromazine equivalents dose equal or below, or above, the mean effective daily dose (ED50) of chlorpromazine as defined previously [28]. Benzodiazepine anxiolytic dose was similarly coded as 0, 1 or 2, with reference to the midpoint of the *Physician’s Desk Reference*-recommended daily dose range for each medication. We generated a composite measure of total medication load, reflecting dose and variety of all different medications taken, by summing all individual medication codes for each medication category for each individual participant. In order to investigate possible effects of the medication load on the results from sample 2, a Pearson product-moment correlation coefficient was computed to assess the relationship between the medication load of each individual and the predictive probabilities found to the patterns of brain response to (1) neutral and (2) happy faces.

#### Pattern classification analysis

We used Gaussian Process Classifier (GPC) [29], a machine learning approach that, in the context of neuroimaging, assigns a predictive probability to an individual pattern of brain activation based on the confidence of a classifier computed from pre-processed fMRI scans. The GPC gives predictive probabilities for stimuli of class 1 and class 2, and by applying a threshold to those probability values we can compute the mean accuracy. The predictive probability gives a measure of how confident the classifier is about the class membership of the test pattern (i.e. pattern of whole brain activation for the test subject). If the predictive probability is close to 0.5, it means that the classifier is not very confident, indicating that the pattern of brain activation being tested is not able to discriminate between the two stimulus classes. On the other hand, if the predictive probability is close to one (or zero), it means that the classifier is confident about the pattern’s class membership, which in turn indicates that the pattern being tested is consistent with the training data. For a detailed description about the GPC implementation to fMRI based classification please see [30]. In order to maximize the differences between the patterns used to train the GPC we only used patterns of brain activation to prototypical emotional (100% sad or 100% happy) and to neutral faces in the current analysis. The rationale here was to obtain a "reference template" for discriminating between emotional vs. neutral brain patterns based on less ambiguous stimuli. Specifically, the images corresponding to the GLM coefficients (sample 1∶100% sad and neutral; sample 2∶100% happy and neutral) defined the spatial patterns of brain activation used as input to the GPC. We used the GPC as implemented in PROBID software (http://www.brainmap.co.uk/PROBID) and additional MATLAB customized codes to enable the novel analysis framework.

**Figure 1:**
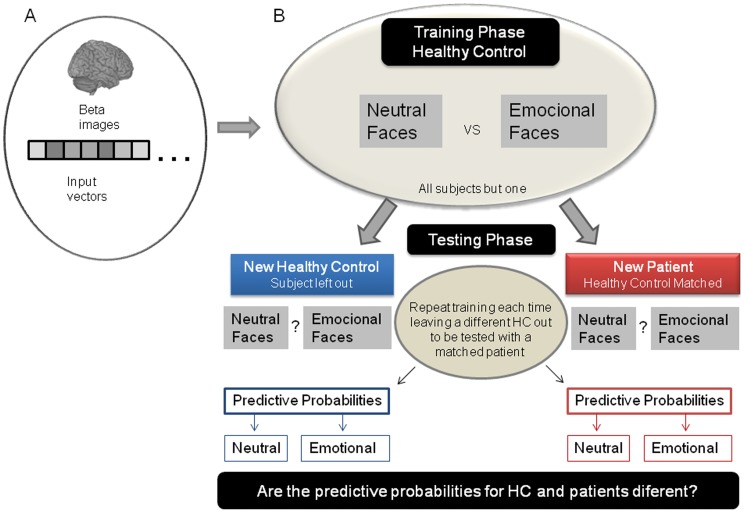
Summary of the pattern recognition analyses. (A) Feature Extraction: the beta images were transformed into an input vector. (B) New pattern recognition framework: we first trained the classifier using data from all but one healthy control subject (by a leave-one-out procedure) to discriminate between brain patterns of activation to emotional (100% sad or 100% happy) from neutral faces. We then tested the classifier using data from the healthy control left out and a gender and age matched depressed patient. Finally, we compared the predictive probabilities between healthy controls and depressed patients.

The analytic framework consisted of two phases: In the first stage, we trained the classifier to discriminate between two brain states (e.g. viewing sad faces vs. viewing neutral faces) using data only from *healthy controls*. In the second stage, we tested the classifier using pattern brain of activations of a healthy control and a patient for the same stimuli. Specifically, we trained the GPC using data from all but one control subject (by a leave-one-out procedure) to discriminate between brain patterns of activation to emotional and to neutral faces. We then tested the classifier using data from the healthy control subject left out and a matched depressed patient (see Figure 1). This procedure was repeated, each time leaving a different healthy control subject out. The aim was to investigate if the patients’ patterns of brain activation would be classified with the same confidence as the healthy controls’ patterns, or if they would be classified with lower confidence, which would suggest that the pattern of brain activations for depressed patients does not resemble the pattern of controls. Finally, we compared the predictive probabilities for the brain activation patterns to emotional and neutral faces between healthy controls and depressed patients using planned t-tests.

#### Permutation test

Permutation test was used to compute statistical significance in two situations in this study. First, this test was used to derive a p-value to determine whether classification accuracy exceeded chance levels (50%). To achieve this, we permuted each class’ labels 1000 times (i.e., each time randomly assigning class 1 and class 2 labels to each pattern of brain activation) and repeated the entire procedure. We then counted the number of times the permuted test accuracy was higher than the one obtained for the true labels. Dividing this number by 1000 we derived a p-value for the classification accuracies.

Permutation test was also used to derive a p-value for the mean difference in the predictive probabilities between healthy controls and depressed patients to emotional and neutral faces. For that we permuted the labels between healthy controls and depressed patients 1000 times (i.e. each time randomly assigning healthy control and depressed patient to each predictive probability obtained to neutral or emotional faces classification). We then counted the number of times the permuted mean differences (healthy controls versus depressed patients) were higher than the one obtained for the true labels. The p-value was derived dividing this number by 1000.

## Results

### Discrimination between Patterns of Brain Activation for Emotional vs. Neutral Faces

For each group (in samples 1 and 2), we trained a GPC using only the HC data to discriminate between the following stimulus contrasts: 100% sad vs. neutral (sample 1) and 100% happy vs. neutral (sample 2). The GPC (based on HC) was then applied to classify patterns of whole brain activation for different facial expression in both groups (HC and DP). The accuracies for classifying patterns of brain activation to emotional and neutral faces were significantly above chance level for both groups of healthy controls. However, for the depressed groups, the GPC accuracies were only significantly above the chance for classifying patterns of brain activation to emotional but not to neutral faces (see Table 2). It is interesting to note that the classifiers were less confident about classifying brain activations to neutral faces specially for depressed patients group as the emotional accuracy (i.e. the percentage of cases for which whole-brain activation to emotional faces were correctly classified as emotional stimulus class) was consistently higher than neutral accuracy (i.e. the percentage of cases for which whole-brain activation to neutral faces were correctly classified as neutral stimulus class). The confusion matrices are showed in the Supplementary Material (Tables S2, S3, S4, S5).

**Table 2:** Within-group decoding accuracy in Healthy Controls (HC) and Depressed Patients (DP).

Contrast	Group	N	Accuracy*	Emotional Accuracy	Neutral Accuracy	p-value
Sad vs. Neutral	HC	19	0.74	0.84	0.63	0.003
	DP	19	0.58	0.95	0.21	0.097
Happy vs. Neutral	HC	18	0.70	0.78	0.61	0.001
	DP	18	0.53	0.78	0.28	0.282

* Overall Accuracy is the mean between emotional accuracy (emotional correctly classified as emotional) and neutral accuracy (neutral correctly classified as neutral).

### Between-group Differences in Predictive Probabilities

**Figure 2:**
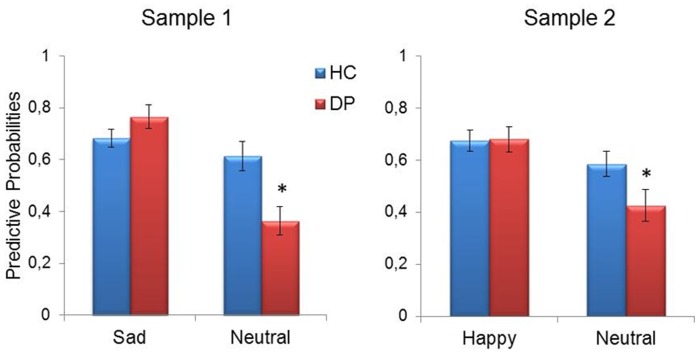
Results from Gaussian Process Classifier (GPC). Comparison of the predictive probabilities to patterns of brain activation to emotional and neutral faces between healthy controls and depressed patients. Note that the predictive probabilities to neutral faces in patients were significantly lower in comparison to the healthy controls. The data are presented with the mean and standard error to the mean. *p<0.05.

The main goal of the present study was to test whether the classifier’s predictions (predictive probabilities) for patterns of brain activation to emotional and neutral faces were different for healthy controls and depressed patients. Interestingly, permutation tests indicated that the predictive probabilities to neutral faces in the patients were significantly lower in comparison to the healthy controls in both samples (sample 1: p = 0.006; sample 2: p = 0.041). These differences were specific to neutral faces. For sad faces (sample 1) and happy faces (sample 2), there were no significant differences in the predictive probabilities between depressed patients and healthy controls (sample 1: p = 0.17; sample 2: p = 0.93) (Figure 2).

In order to explore the possible contributions of the medication load to explain the results found in sample 2, we performed Person correlation analyses between medication load and the predictive probabilities to neutral and happy faces. There was no significant correlation between medication load and predictive probabilities to neutral faces (r = 0.33, n = 18, p = 0.22) nor between medication load and predictive probabilities to happy faces (r = −0.18, n = 18, p = 0.48).

## Discussion

Our results suggest that the pattern of brain activation to neutral faces in depressed patients is not consistent with the pattern of brain activation in healthy subjects to the same stimuli. In the present study we investigated whether the behavioural misclassification of neutral faces previously reported in depressed patients [11], [12] could be also observed at brain network level measured by fMRI. To address this question, we applied a novel pattern recognition framework to fMRI data of two independent samples of depressed patients and healthy controls. First, a Gaussian Process Classifier was trained to discriminate between patterns of brain activation to emotional and neutral faces using data only from healthy controls. Second, the classifier was tested using data from a new healthy control and a matched patient. Finally, we applied the post-hoc tests, to examine whether the predictive probabilities to patterns of brain activation to neutral or to emotional faces were significantly different between groups.

We found that the predictive probabilities to patterns of brain activation to neutral faces in all patient groups were significant lower in comparison to the healthy controls. This result was specific to neutral faces, i.e. there were no significant differences between the groups when considering the predictive probabilities to patterns of brain activation to sad faces (sample 1) and to happy faces (samples 2). The predictive probability measures the classifier’s confidence about the class membership of a test example. Therefore, these findings suggest that the pattern of brain activation to neutral faces in depressed patients was not consistent with the pattern of brain activation to neutral faces in healthy controls. These results are in agreement with the behavioural studies demonstrating that depressed patients label neutral faces as neutral with significantly less accuracy than either happy or sad faces [11] and they are slower to respond to neutral than emotional expressions [11], [12]. Similarly, we have recently shown that patterns of brain activation to neutral faces could help to differentiate healthy adolescents genetically at-risk for bipolar disorder from healthy adolescents at low risk of developing these disorders [20]. Interestingly, a carefully inspection in table 2 indicate that the accuracy to classify neutral stimuli as neutral was much lower than the accuracy to classify emotional stimuli as emotional in depressed patients, suggesting a bias to classify neutral stimuli as emotional in these patients. In order to investigate whether this bias depends on the novel framework of this study, i.e. training the classifier using only patterns of brain activity from Healthy Controls, we performed additional analyses within each group independently (i.e. training with Healthy Controls or Depressed Patients - Supplemental methods (Text S1) and Table S1). The GPC was able to accurately discriminate between the patterns of brain activity for emotional expressions versus neutral in both samples and the bias to consider neutral stimuli as emotional is less clear, seems to occur only in sample 2. Then, the results of our framework indicate that for both samples the patterns of activity to neutral expressions in depressed patients is not consistent with the patterns of healthy subjects to the same stimuli (as suggested by the lower classification accuracy for neural faces). Thus, we suggest that the proposed framework applied in the present study, i.e. using patterns of brain activity from Healthy Controls as a “reference pattern”, reveals the importance of brain responses to neutral faces in depressed patients.

Taken together, these results support the hypothesis that depressed individuals may interpret emotionally neutral cues as emotionally meaningful [1]. Neutral faces are often perceived as ambiguous and potentially threatening by individuals diagnosed with a depression or mood disorder [1], [31]. For instance, one study reported abnormally elevated subcortical activation to neutral faces in youth with bipolar type I disorder, particularly in those who perceived these faces as threatening [31]. This fits with findings of earlier research that indicates that depressed individuals are more likely to interpret neutral faces negatively [11], [32], [33]. Further, this is also consistent with findings of enhanced memory for negative material in memory tasks in major depression [34].

It is interesting to note that findings were consistent across two independent samples of healthy controls and depressed patients studied, suggesting the robustness of our findings. This is an important point because pattern recognition analyses generally aim to develop robust algorithms to identify differences between classes of interest that are independent of variables of no interest (for instance, differences related to different scanners, acquisition protocol, etc). Furthermore, correlation analyses showed that the classifier’s predictions were not correlated with medication load (i.e., sample 1 was a free-medication sample and the medication load of sample 2 was not significantly associated with the GPC predictive probabilities), although, for the sample 2 we cannot fully discard some influence in the results caused by the medication load. In fact, the results for sample 2 were less robust than those for sample 1, suggesting that medication load could have been a confounding variable. Our work differs from previous applications of pattern recognition approaches to patient classification based on fMRI data as its main goal was not to directly discriminate the groups. Our aim was to use pattern recognition approaches to test a hypothesis about differences in face expression processing between healthy controls and patients. One advantage of using pattern recognition in this context is the ability to investigate differences in brain processing at a network level, i.e. analysing the whole pattern of brain activation. One possible clinical application of this framework could be training a classifier to discriminate between emotional and neutral stimuli using a large normative basis of healthy control subjects. This analysis would define a “healthy” discrimination between patterns of brain activation to emotional vs. neutral stimuli. One could then apply the classifier to a new subject as a diagnostic approach. If the classifiers’ predictive probability to neutral stimuli would be below a validated threshold, there would be evidence that the subject’s pattern of brain activation was different from the healthy controls pattern which would indicate that the subject was a patient.

A limitation of this study is that the predictive probabilities of the healthy control groups were obtained using a leave-one-out framework and the predictive probabilities of the patient groups were obtained using an independent sample (the patients were never used to train the classifier). The leave-one-out framework is an unbiased approach for assessing how the results of a classifier will generalize to an independent data set. On the other hand, it should be noted that the predictive probabilities for each matched pair of healthy control and patient are based on the same classifier (which excludes the test healthy control from the training). Ideally the classifier should be trained with one sample of healthy control and tested with independent samples of healthy controls and patients.

In summary, we showed that the misinterpretation of neutral faces as emotionally-salient in depressed individuals may have a neural substrate. The lower confidence of the GPC in classifying patterns of brain activity to neutral faces in depressed patients when compared with HC patterns suggests that the depressed patients might engage a different brain network when processing the neutral stimuli or there might be more variability in the brain network engaged by the patients. These results also suggest that examination of brain activation to neutral faces can provide insights about pathophysiologic processes in depression on an individual-level, case-by-case basis.

## Supporting Information

Table S1 **Within-group decoding accuracy using the “standard” pattern recognition framework, i.e., training with HC and DP separately to make predictions to HC and DP, respectively.**
(DOC)

Table S2 **Confusion matrix to discrimination Sad versus Neutral in the Healthy Control sample.**
(DOC)

Table S3 **Confusion matrix to discrimination Sad versus Neutral in the Depression Patients sample.**
(DOC)

Table S4 **Confusion matrix to discrimination Happy versus Neutral in the Healthy Control sample.**
(DOC)

Table S5 **Confusion matrix to discrimination Happy versus Neutral in the Depression Patients sample.**
(DOC)

Text S1 **Supplemental Information.**
(DOC)

## References

[1] Bourke C, Douglas K, Porter R (2010) Processing of facial emotion expression in major depression: a review. Aust N Z J Psychiatry 44: 681–696. 20636189 10.3109/00048674.2010.496359

[2] Phillips ML, Drevets WC, Rauch SL, Lane R (2003) Neurobiology of emotion perception II: Implications for major psychiatric disorders. Biol Psychiatry 54: 515–528. 12946880 10.1016/s0006-3223(03)00171-9

[3] Lawrence NS, Williams AM, Surguladze S, Giampietro V, Brammer MJ, et al (2004) Subcortical and ventral prefrontal cortical neural responses to facial expressions distinguish patients with bipolar disorder and major depression. Biol Psychiatry 55: 578–587. 15013826 10.1016/j.biopsych.2003.11.017

[4] Surguladze S, Brammer MJ, Keedwell P, Giampietro V, Young AW, et al (2005) A differential pattern of neural response toward sad versus happy facial expressions in major depressive disorder. Biol Psychiatry 57: 201–209. 15691520 10.1016/j.biopsych.2004.10.028

[5] Fu CH, Williams SC, Cleare AJ, Brammer MJ, Walsh ND, et al (2004) Attenuation of the neural response to sad faces in major depression by antidepressant treatment: a prospective, event-related functional magnetic resonance imaging study. Arch Gen Psychiatry 61: 877–889. 15351766 10.1001/archpsyc.61.9.877

[6] Fu CH, Williams SC, Brammer MJ, Suckling J, Kim J, et al (2007) Neural responses to happy facial expressions in major depression following antidepressant treatment. Am J Psychiatry 164: 599–607. 17403973 10.1176/ajp.2007.164.4.599

[7] Stuhrmann A, Suslow T, Dannlowski U (2011) Facial emotion processing in major depression: a systematic review of neuroimaging findings. Biol Mood Anxiety Disord 1: 10. 22738433 10.1186/2045-5380-1-10 PMC3384264

[8] Almeida JR, Versace A, Mechelli A, Hassel S, Quevedo K, et al (2009) Abnormal amygdala-prefrontal effective connectivity to happy faces differentiates bipolar from major depression. Biol Psychiatry 66: 451–459. 19450794 10.1016/j.biopsych.2009.03.024 PMC2740996

[9] Cullen KR, Gee DG, Klimes-Dougan B, Gabbay V, Hulvershorn L, et al (2009) A preliminary study of functional connectivity in comorbid adolescent depression. Neuroscience letters 460: 227–231. 19446602 10.1016/j.neulet.2009.05.022 PMC2713606

[10] Jiao Q, Ding J, Lu G, Su L, Zhang Z, et al (2011) Increased Activity Imbalance in Fronto-Subcortical Circuits in Adolescents with Major Depression. PLoS ONE 6(9): e25159 doi:10.1371/journal.pone.0025159. 21949877 10.1371/journal.pone.0025159 PMC3175001

[11] Leppänen JM, Milders M, Bell JS, Terriere E, Hietanen JK (2004) Depression biases the recognition of emotionally neutral faces. Psychiatry Res 128: 123–133. 15488955 10.1016/j.psychres.2004.05.020

[12] Suslow T, Dannlowski U, Lalee-Mentzel J, Donges US, Arolt V, et al (2004) Spatial processing of facial emotion in patients with unipolar depression: a longitudinal study. J Affect Disord 83: 59–63. 15546646 10.1016/j.jad.2004.03.003

[13] Mourão-Miranda J, Bokde AL, Born C, Hampel H, Stetter M (2005) Classifying brain states and determining the discriminating activation patterns: Support Vector Machine on functional MRI data. Neuroimage 28: 980–995. 16275139 10.1016/j.neuroimage.2005.06.070

[14] Haynes JD, Rees G (2006) Decoding mental states from brain activity in humans. Nat Rev Neurosci 7: 523–534. 16791142 10.1038/nrn1931

[15] Norman KA, Polyn SM, Detre GJ, Haxby JV (2006) Beyond mind-reading: multi-voxel pattern analysis of fMRI data. Trends Cogn Sci 10: 424–430. 16899397 10.1016/j.tics.2006.07.005

[16] Fu CH, Mourao-Miranda J, Costafreda SG, Khanna A, Marquand AF, et al (2008) Pattern classification of sad facial processing: toward the development of neurobiological markers in depression. Biol Psychiatry 63: 656–662. 17949689 10.1016/j.biopsych.2007.08.020

[17] Ecker C, Marquand A, Mourão-Miranda J, Johnston P, Daly EM, et al (2010) Describing the brain in autism in five dimensions–magnetic resonance imaging-assisted diagnosis of autism spectrum disorder using a multiparameter classification approach. J Neurosci 30: 10612–10623. 20702694 10.1523/JNEUROSCI.5413-09.2010 PMC6634684

[18] Hahn T, Marquand AF, Ehlis AC, Dresler T, Kittel-Schneider S, et al (2011) Integrating neurobiological markers of depression. Arch Gen Psychiatry 68: 361–368. 21135315 10.1001/archgenpsychiatry.2010.178

[19] Mourão-Miranda J, Almeida JR, Hassel S, de Oliveira L, Versace A, et al (2012) Pattern recognition analyses of brain activation elicited by happy and neutral faces in unipolar and bipolar depression. Bipolar Disord 14: 451–460. 22631624 10.1111/j.1399-5618.2012.01019.x PMC3510302

[20] Mourão-Miranda J, Oliveira L, Ladouceur CD, Marquand A, Brammer M, et al (2012) Pattern recognition and functional neuroimaging help to discriminate healthy adolescents at risk for mood disorders from low risk adolescents. PLoS One 7: e29482. 22355302 10.1371/journal.pone.0029482 PMC3280237

[21] Friston KJ, Holmes AP, Worsley KJ, Poline JP, Frith CD, et al (1994) Statistical parametric maps in functional imaging: A general linear approach. Hum Brain Mapp. 2(4): 189–210.

[22] First MB, Spitzer RL, Gibbon M, Williams JBW (1995) Structured Clinical Interview for DSM-IV Axis I Disorders. New York: New York State Psychiatric. Institute, Biometrics Research.

[23] Hamilton M (1960) A rating scale for depression. J Neurol Neurosurg Psychiatry 23: 56–62. 14399272 10.1136/jnnp.23.1.56 PMC495331

[24] Lishman WA (1994) American Psychiatric Association. Diagnostic and statistical manual of mental disorders : DSM-IV. 4th ed. Washington, D.C.: American Psychiatric Association.

[25] Ekman P, Freisen WV (1976) Pictures of Facial Affect. Palo Alto, CA: Consulting Psychologists Press.

[26] Gilbert AR, Mataix-Cols D, Almeida JR, Lawrence N, Nutche J, et al (2008) Brain structure and symptom dimension relationships in obsessive-compulsive disorder: a voxel-based morphometry study. J Affect Disord 109: 117–126. 18342953 10.1016/j.jad.2007.12.223

[27] Versace A, Almeida JR, Hassel S, Walsh ND, Novelli M, et al (2008) Elevated left and reduced right orbitomedial prefrontal fractional anisotropy in adults with bipolar disorder revealed by tract-based spatial statistics. Arch Gen Psychiatry 65: 1041–1052. 18762590 10.1001/archpsyc.65.9.1041 PMC2730162

[28] Davis JM, Chen N (2004) Dose response and dose equivalence of antipsychotics. J Clin Psychopharmacol 24: 192–208. 15206667 10.1097/01.jcp.0000117422.05703.ae

[29] Rasmussen C, Williams CKI (2006) Gaussian Processes for Machine Learning. Cambridge, Massachusetts: The MIT Press.

[30] Marquand A, Howard M, Brammer M, Chu C, Coen S, et al (2010) Quantitative prediction of subjective pain intensity from whole-brain fMRI data using Gaussian processes. Neuroimage 49: 2178–2189. 19879364 10.1016/j.neuroimage.2009.10.072

[31] Rich BA, Vinton DT, Roberson-Nay R, Hommer RE, Berghorst LH, et al (2006) Limbic hyperactivation during processing of neutral facial expressions in children with bipolar disorder. Proc Natl Acad Sci U S A 103: 8900–8905. 16735472 10.1073/pnas.0603246103 PMC1482675

[32] Gur RC, Erwin RJ, Gur RE, Zwil AS, Heimberg C, et al (1992) Facial emotion discrimination: II. Behavioral findings in depression. Psychiatry Res 42: 241–251. 1496056 10.1016/0165-1781(92)90116-k

[33] Gollan JK, Pane HT, McCloskey MS, Coccaro EF (2008) Identifying differences in biased affective information processing in major depression. Psychiatry Res 159: 18–24. 18342954 10.1016/j.psychres.2007.06.011 PMC2571942

[34] Matt GE, Vazquez C, Campbell WK (1992) Mood-congruent recall of affectively toned stimuli: a meta-analytic review. Clin Psychol Rev 12: 227–255.

