# Supplementary material for: What Does Brain Response to Neutral Faces Tell Us about Major Depression? Evidence from Machine Learning and fMRI
Source: PLoS One. 2013 Apr 1;8(4):e60121. doi: 10.1371/journal.pone.0060121 (PMC3613341; doi:10.1371/journal.pone.0060121)
Supplement: Table S1 — Within-group decoding accuracy using the “standard” pattern recognition framework, i.e., training with HC and DP separately to make predictions to HC and DP, respectively. (DOC) [file pone.0060121.s001.doc]

| **Contrast** | N per group | **Group** | **Accuracy** | **Emotional correctly classified** | **Neutral correctly classified** | **p-value** |
| --- | --- | --- | --- | --- | --- | --- |
| Sample 1  sad x neutral  Sample2  happy x neutral | 19 | HC | 0.81 | 1.0 | 0.63 | 0.001 |
|  | 19 | DP | 0.63 | 0.58 | 0.68 | 0.033 |
|  | 18 | HC | 0.81 | 0.94 | 0.72 | 0.001 |
|  | 18 | DP | 0.70 | 0.89 | 0.50 | 0.002 |
