# Supplementary material for: What Does Brain Response to Neutral Faces Tell Us about Major Depression? Evidence from Machine Learning and fMRI
Source: PLoS One. 2013 Apr 1;8(4):e60121. doi: 10.1371/journal.pone.0060121 (PMC3613341; doi:10.1371/journal.pone.0060121)
Supplement: Table S2 — Confusion matrix to discrimination Sad versus Neutral in the Healthy Control sample. (DOC) [file pone.0060121.s002.doc]

|  | **Sad**  **(predict)** | **Neutral**  **(predict)** |
| --- | --- | --- |
| **Sad (actual)** | 84 | 37 |
| **Neutral (actual)** | 16 | 63 |
