# Supplementary material for: What Does Brain Response to Neutral Faces Tell Us about Major Depression? Evidence from Machine Learning and fMRI
Source: PLoS One. 2013 Apr 1;8(4):e60121. doi: 10.1371/journal.pone.0060121 (PMC3613341; doi:10.1371/journal.pone.0060121)
Supplement: Text S1 — Supplemental Information. (DOC) [file pone.0060121.s006.doc]

SUPPLEMENTAL INFORMATION

Supplemental Methods

Within-group stimuli classification (Table S1)

Within each group (HC and DP) we examined the ability of the GPC to accurately discriminate between the patterns of whole brain activity for intensity facial expressions versus neutral. In each group separately we evaluated the performance of GPC to discriminate between neural activity patterns and emotional neural activity using a leave-one-subject-out cross validation test. Here, for each trial we first used data from all but one individual to train the classifier. Then, we predicted the emotion of facial expressions using the brain scans of the remaining individual (one of each class). We used a threshold of 0.5 to decide its class membership, i.e. if the predictive probability was above 0.5 it corresponded to class 1, otherwise (0.5 or less) it corresponded to class 2. The mean classification accuracy was the mean of the true positive (i.e. percentage of emotional stimuli correct classified) and true negative rates (i.e. percentage of neutral stimuli correct classified).

Permutation test

This test was used to derive a p-value to determine whether classification accuracy exceeded chance levels (50%). To achieve this, we permuted the class labels 1000 times (i.e., each time randomly assigning class 1 and class 2 labels to each pattern of brain activation) and repeated the entire RFE procedure. We then counted the number of times the permuted test accuracy was higher than the one obtained for the true labels. Dividing this number by 1000 we derived a p-value for the classification accuracies.
